# Genetic and Epigenetic Diversity of *Salmonella enterica* Isolates from Clinical and Veterinary Sources in Kazakhstan

**DOI:** 10.3390/pathogens15101074

**Published:** 2026-10-09

**Authors:** Duman T. Yessimseit, Altyn K. Rysbekova, Zauresh B. Zhumadilova, Beck Z. Abdeliyev, Nur B. Tukhanova, Altynai K. Kassenova, Aigul K. Abdrakhmanova, Akmaral Mereke, Sanzhar D. Agzam, Ainur S. Nurpeisova, Raikhan Nissanova, Ayaulym M. Maksatova, Oleg N. Reva, Aigul A. Abdirassilova

**Affiliations:** 1 M. Aikimbayev’s National Scientific Center for Especially Dangerous Infections, 14 Zhakhanger St., Almaty A35P0K3, Kazakhstan; yessimseit@gmail.com (D.T.Y.); rysbekova23@mail.ru (A.K.R.); zzbgdirect@nscedi.kz (Z.B.Z.); abdelbeck@gmail.com (B.Z.A.); tukhanovanur@gmail.com (N.B.T.); kassenovaaltynai@gmail.com (A.K.K.); sanzhar.dauletuly.agzam@gmail.com (S.D.A.); a.maksatova@nscedi.kz (A.M.M.); 2 Department of Molecular Biology and Genetics, Faculty of Biology and Biotechnology, Al-Farabi Kazakh National University, 71 al-Farabi Ave., Almaty A15E3B4, Kazakhstan; 3 I. Zhekenova’s City Clinical Infectious Disease Hospital, Almaty A36G2X0, Kazakhstan; a.kamet@mail.ru (A.K.A.); akmaral.mereke@bk.ru (A.M.); 4 Kazakh Scientific Research Veterinary Institute, 223 Rayymbek Ave., Almaty A20C2E4, Kazakhstan; nurai1005@gmail.com (A.S.N.); raihan.nisanova@gmail.com (R.N.); 5 Centre for Bioinformatics and Computational Biology, Department of Biochemistry, Genetics and Microbiology, University of Pretoria, Pretoria 0002, South Africa

**Keywords:** *Salmonella enterica*, genome, virulence, antibiotic resistance, plasmid, epigenetics

## Abstract

*Salmonella enterica* is a major cause of foodborne and invasive infections worldwide. This retrospective study used whole-genome sequencing to compare the genotypes and characterize the genomic diversity of bacterial strains preserved in an archived culture collection, originating from clinical and veterinary sources and predominantly from Kazakhstan. Twenty-two bacterial isolates were analyzed by Illumina whole-genome sequencing; subsequent characterization confirmed twenty as *S. enterica* and identified two as *Escherichia coli*. Six selected *S. enterica* strains representing different lineages were additionally sequenced using PacBio Revio SMRT technology for DNA methylation profiling. Genome assembly, plasmid reconstruction, MLST genotyping, and analyses of virulence genes, antimicrobial resistance determinants, restriction-modification (R-M) operons, and orphan methyltransferases were performed. Comparative genomic analysis revealed substantial genotypic diversity within the collection, while phylogenetic grouping was consistent with MLST classification. ST11 was the most represented sequence type among the selected strains, comprising 11 of the 20 *S. enterica* isolates, including all three recent clinical *S. enterica* isolates. All six PacBio-sequenced genomes showed >99% DAM-associated adenine methylation at GATC motifs, while DCM-mediated cytosine methylation occurred at approximately 35% of CCWGG motifs. Adenine methylation at CAGAG is controlled by additional type III R-M systems. Strain 19S showed a distinct type I-associated adenine methylation pattern together with several HsdM amino acid substitutions; however, the functional relationship between these observations requires experimental validation. The study demonstrates the value of retrospective genomic characterization of culture collections for revealing genetic and epigenetic diversity preserved among historical and recent bacterial isolates and provides a basis for future population-level and functional studies.

## 1. Introduction

According to the World Health Organization (WHO), foodborne infections caused by bacteria of the genus *Salmonella* remain a major public health concern worldwide because of their broad host range, high transmission potential, and increasing levels of antimicrobial resistance (AMR) [1]. The expectation that climate warming may promote the widespread dissemination of antibiotic-resistant pathogens has recently been supported by studies using *Salmonella* as a model organism [2]. This finding highlights the need to establish surveillance measures for dynamic monitoring of changes in pathogen populations and the emergence of new unusual genotypes.

Members of this genus belong to the family Enterobacteriaceae and are currently classified into two species, *Salmonella bongori* and *Salmonella enterica* [3]. Among them, *S. enterica* is responsible for the overwhelming majority of infections in humans and warm-blooded animals. The species is highly heterogeneous and includes numerous lineages adapted to different ecological niches and transmission strategies [4].

Within *S. enterica*, the subspecies enterica contains most clinically important isolates and is further subdivided into a large number of serovars based on combinations of somatic and flagellar antigens together with biochemical characteristics [5]. These serovars are traditionally divided into typhoidal and non-typhoidal groups according to their host specificity and disease manifestations [6,7]. Typhoidal *Salmonella* (TS), represented mainly by *S. enterica* serovars Typhi and Paratyphi A, B, and C, are highly specialized human-adapted pathogens responsible for enteric fever. Transmission occurs predominantly through the fecal-oral route via contaminated food or drinking water, although direct person-to-person transmission may also occur. After entering the host, these pathogens can evade early immune recognition and establish prolonged systemic infections characterized by persistent fever and bacteremia [5].

In contrast, non-typhoidal *Salmonella* (NTS) exhibit a broader host range and are commonly associated with zoonotic transmission. In most cases, NTS infections manifest as self-limiting gastroenteritis; however, some strains are capable of causing severe invasive disease [8]. These invasive non-typhoidal *Salmonella* (iNTS) strains penetrate the intestinal barrier and disseminate into the bloodstream or internal organs, resulting in septicemia and systemic infection. iNTS disease is particularly dangerous in infants, elderly individuals, malnourished patients, immunocompromised persons, and people living with HIV [9]. Mortality associated with bloodstream infections caused by iNTS remains high in many regions of the world [10]. Among the serovars frequently associated with invasive disease, *S. enterica* serovar Typhimurium is one of the most highly reported [11].

The introduction of molecular epidemiological approaches, particularly Multi-Locus Sequence Typing (MLST), has revealed extensive diversity within major *Salmonella* serovars [12]. MLST analysis demonstrated that strains belonging to the same serovar may form multiple clonal lineages differing in geographical distribution, host adaptation, virulence potential, and antimicrobial resistance profiles [13,14]. Some sequence types (STs) are globally disseminated, whereas others appear to be restricted to specific ecological or geographical regions. For example, *S. enterica* Typhimurium ST19 is considered one of the most widespread and evolutionarily generalized lineages, commonly associated with zoonotic transmission from livestock to humans [15]. In contrast, ST34 strains are frequently characterized by broader antimicrobial resistance profiles, including multidrug resistance, and have become increasingly associated with intensive animal production systems and foodborne transmission [16,17].

Regional studies indicate that the population structure of *Salmonella* may differ substantially between countries and epidemiological settings [14]. A recent genomic study characterized poultry-derived *S. enterica* isolates from southern Kazakhstan, many of which were ST11 variants [18]. Nevertheless, the evolutionary relationships, virulence-associated genomic features, and epigenetic characteristics of these circulating strains remain insufficiently characterized.

Advances in third-generation sequencing technologies, including PacBio Single-Molecule Real-Time (SMRT) sequencing technology and Oxford Nanopore sequencing technology, now enable not only high-quality genome assembly but also direct detection of epigenetic modifications such as DNA methylation [19]. These approaches provide new opportunities to investigate the roles of restriction–modification systems, methyltransferases (MTases), horizontal gene transfer, and epigenetic regulation in the adaptation and pathogenicity of *Salmonella* strains [20,21].

Nucleotide methylation occurs through the activity of MTase, which are often components of restriction-modification (R-M) systems that protect bacterial cells from bacteriophages and conjugative plasmids. However, orphan MTases are also found, and their role in the biology of pathogenic microorganisms remains incompletely understood. It was reported before that gnomic DNA methylation in *Salmonella* occurred in both adenine and cytosine residues at versatile sequence motifs recognized by MTases [20]. Scientific evidence indicates that genomic DNA methylation represents one of the mechanisms regulating gene expression and has a substantial influence on pathogenesis, the development of antibiotic resistance, and the ability of pathogens to survive in the environment [20,21,22].

The present study aimed to characterize the genetic and epigenetic diversity preserved in an archived collection of *S. enterica* isolates obtained from clinical and veterinary sources predominantly in the Almaty region of Kazakhstan over different time periods. While recent genomic studies have characterized particular circulating *Salmonella* populations, considerably less information is available on the genomic and epigenetic diversity represented in historical bacterial collections. We therefore selected strains originating from different collection periods and independent clinical or veterinary episodes to obtain a diverse set of isolates and to avoid intentional enrichment for particular genotypes or phenotypes. Whole-genome sequencing was used to determine genetic relationships, genotype frequencies, plasmid composition, and the distribution of virulence- and AMR-associated determinants, while selected strains were additionally analyzed by PacBio sequencing to characterize DNA methylation patterns. The study was designed primarily to characterize the diversity preserved within the collection rather than to establish associations between particular genotypes and clinical, virulence, or AMR phenotypes.

## 2. Materials and Methods

### 2.1. Study Design and Ethics Approval

A retrospective study of an archived collection of bacterial isolates was conducted. The study protocol and related materials were reviewed by the Local Ethics Committee of the M. Aikimbayev National Scientific Center for Especially Dangerous Infections (NSCEDI, Almaty, Kazakhstan; Protocol No. 2 dated 12 February 2024).

Clinical isolates had originally been obtained during routine laboratory diagnostics and infectious-disease surveillance and were not collected specifically for the present study. Archived bacterial cultures and associated laboratory data were made available to the researchers by the National Collection of Microorganisms (NCM) at NSCEDI on 9 February 2026. Before the materials were provided for analysis, all patient-identifying information had been removed. The study involved no direct patient participation, contact, study-specific intervention, or additional collection of clinical specimens.

### 2.2. Collection of Bacterial Isolates

The study included 22 archived bacterial isolates maintained in the National Collection of Microorganisms (NCM) at NSCEDI. The collection comprised historical isolates together with four recent clinical isolates originally obtained from the I. Zhekenova City Clinical Infectious Diseases Hospital (CCIDH, Almaty, Kazakhstan) and four recent animal-derived isolates provided by the Kazakh Scientific Research Veterinary Institute (KSRVI).

The isolates were selected from the available collection to represent different collection periods and independent clinical or veterinary isolation episodes rather than according to a particular serotype, genotype, virulence profile, or AMR phenotype for recent clinical isolates. This selection strategy was used to reduce intentional enrichment for particular genetic or phenotypic characteristics and to provide a heterogeneous set of collection strains for comparative genomic analysis. Inclusion required the availability of a viable archived bacterial culture suitable for recovery and DNA isolation. No isolates were excluded on the basis of their expected genotype or resistance phenotype.

The investigated strains were deposited in the respective collections between 1957 and 2025. The collection included human- and animal-derived isolates of diverse geographic origin, predominantly from regions of Kazakhstan, and encompassed both commonly represented serovars and less common genetic lineages. The isolates had previously been preliminarily identified as members of the genus *Salmonella* during routine diagnostic activities based on cultural, morphological, and biochemical characteristics conventionally used for differentiation of *Salmonella* from other Enterobacterales. Isolates were examined by Gram staining and characterized using Kligler iron agar and additional biochemical tests. A typical *Salmonella* profile included glucose fermentation with an acid butt and alkaline slant, absence of lactose and sucrose fermentation, frequent H_2_S and gas production, negative urease and indole reactions, nitrate reduction to nitrite, and absence of gelatin hydrolysis [23]. The results of the preliminary identification were obtained from the collection records, and the biochemical tests were not repeated in the present study.

Source, geographic origin, and the available date of deposit of the selected isolates are presented in Table 1.

### 2.3. Recovery and Cultivation of Bacterial Isolates

Clinical isolates were recovered by personnel of the bacteriological laboratory of the infectious disease hospital during routine diagnostic testing and were subsequently deposited in the National Collection of Microorganisms for long-term preservation. Animal-derived isolates were obtained during routine veterinary diagnostic activities conducted in accordance with applicable biosafety and bioethical requirements.

All bacterial isolates were stored at −80 °C in medium containing 20% glycerol. Before laboratory analysis, cryopreserved cultures were recovered in Luria–Bertani (LB) broth containing 10 g/L tryptone, 5 g/L yeast extract, and 5 g/L NaCl. Cultures were incubated at 37 °C for 12 h with continuous agitation on an orbital shaker.

After recovery, cultures were streaked onto Salmonella–Shigella agar (SS agar; HiMedia Laboratories Pvt. Ltd., Mumbai, India) prepared according to the manufacturer’s instructions; the final pH of the medium was 7.2. Plates were incubated aerobically at 37 °C for 18–24 h. After incubation, bacterial growth and colony morphology were assessed. Individual colonies were subcultured onto meat-peptone agar (MPA) to obtain pure cultures.

Pure cultures were grown on Hottinger agar and MPA at 37 °C for 18–24 h. Cultural characteristics were assessed visually according to colony shape, size, transparency, surface characteristics, pigmentation, density, and uniformity. Morphological characteristics of bacterial cells were evaluated by light microscopy after Gram staining.

### 2.4. Serological Identification

Serological identification of the investigated isolates was performed using the agglutination reaction with polyvalent *Salmonella* O-antisera of groups A–E and mono-receptor *Salmonella* O- and H-antisera, in accordance with methodological guideline MU 4.2.2723-10, “Laboratory Diagnosis of Salmonellosis and Detection of *Salmonella* in Food Products and Environmental Samples” [24]. Diagnostic antisera produced by the Saint Petersburg Research Institute of Vaccines and Sera and PETSAL (Russia) were used in this study. Serotyping was performed according to standard *Salmonella* serological classification principles based on somatic (O) and flagellar (H) antigen determination following the White-Kauffmann-Le Minor scheme [25].

The reference strain *S. enterica* serovar Typhimurium TA98 (V RCM-0162) from the Russian Collection of Microorganisms (RCM) was used as a control microorganism in all experimental series and served as a reference control strain.

### 2.5. DNA Isolation and Whole-Genome Sequencing

Genomic DNA was extracted using the QIAamp DNA Mini Kit (Qiagen, Hilden, Germany) according to the manufacturer’s protocol from bacterial cultures grown overnight (for 18 h) at 37 °C on meat-peptone bullion (MPB). To ensure high sample quality and remove potential inhibitors of enzymatic reactions, an additional purification step was performed using AMPure XP magnetic beads (Beckman Coulter, Brea, CA, USA). The integrity and molecular weight of the extracted DNA were assessed by agarose gel electrophoresis. DNA concentration and purity were determined using a NanoDrop 1000 spectrophotometer (Thermo Fisher Scientific, Waltham, MA, USA) and a Qubit 4 fluorometer (Thermo Fisher Scientific, Waltham, MA, USA).

Libraries for whole-genome sequencing were prepared using the tagmentation-based Illumina DNA Prep kit (Illumina, San Diego, CA, USA) with unique dual indexes from the IDT for Illumina DNA/RNA UD Indexes Set A (Integrated DNA Technologies (IDT), Coralville, IA, USA). Sequencing was performed on an Illumina MiSeq platform (Illumina, USA) installed in NSCEDI using the MiSeq Reagent Kit v3 (600 cycles). A total of 17 pM DNA libraries were loaded into the sequencer for each run. Primary processing and quality control of the generated reads were carried out using the instrument’s standard software tools.

Six genomic DNA samples from *S. enterica* strains 1S, 2S, 10S, 14S, 15S, and 19S were sequenced at NSCEDI using Single-Molecule Real-Time (SMRT) sequencing on the PacBio Revio platform (Pacific Biosciences, Menlo Park, CA, USA) for DNA methylation profiling.

### 2.6. Bioinformatics Tools and Statistical Analysis

De novo genome assembly was performed using the assemblers SPAdes v3.15.0 [26] for short Illumina reads and Flye v2.9.6 for long PacBio reads [27]. Plasmid clustering, reconstruction, and typing were carried out using MOB-suite v3.1.9 [28]. Integration of sequencing data and optimization of short-read assembly was performed using the ILLAMINA v1.0 pipeline (https://zenodo.org/records/15227656, accessed on 6 October 2026); and the LRASS v1.0 pipeline (https://zenodo.org/records/16571119, accessed on 6 October 2026) was used for the long-read assembly.

Virulence genes were identified using Abricate v1.4.0 against the VFDB database, and antimicrobial resistance genes were identified using RGI v6.0.5 with the CARD database [29]. Heatmaps were generated in Python-3.14.4 using json-2.0.9, pandas-2.2.3, NumPy-2.3.2, Matplotlib-3.10.1, and SciPy-1.15.2 using the gene presence-absence matrix generated from Abricate and RGI output files. Sample similarity was estimated using the Hamming distance applied to binary feature profiles, followed by hierarchical clustering using average linkage (UPGMA) implemented in scipy.cluster.hierarchy. Matrix rows were reordered according to dendrogram leaf order, and the resulting clustering tree (dendrogram) was plotted adjacent to the heatmap.

Multilocus sequence typing (MLST) was performed based on the PubMLST online database (https://pubmlst.org/organisms/Salmonella-spp, accessed on 6 October 2026) using the MLST scheme based on sequence types of seven core genes: *aroC*, *dnaN*, *hemD*, *hisD*, *purE*, *sucA*, and *thrA*.

Programs ipdSummary and MotifMaker, parts of the software package smrtlink_25.2.0.266456 (https://www.pacb.com/support/software-downloads/, accessed on 6 October 2026) installed on the computer cluster at the Centre for Bioinformatics and Computational Biology (University of Pretoria, Pretoria, South Africa), were used for statistical prediction of epigenetically modified nucleotides and DNA methylation motifs, respectively. For estimation of distribution of methylated sites across coding, non-coding and promoter regions 75 bp upstream of predicted gene start codons, the assembled sequences were annotated using the program Bakta-1.9.4 [30].

A core genome single nucleotide polymorphism (SNP) matrix was generated using Panaroo-1.6.0 [31]. Program MEGA12 [32] was used for protein sequence alignment, identification of mutated loci and clustering proteins using the Neighbor-Joining algorithm.

Detection and analysis of R-M gene clusters in assembled genomes was carried out using DefenseFinder and macsyfinder-2.1.4 [33]. Reconstruction of 3D structures of methyltransferase proteins by their amino acid sequences was conducted on ColabFold v1.6.1: AlphaFold2 Web-based notebook (https://colab.research.google.com/github/sokrypton/ColabFold/blob/main/batch/AlphaFold2_batch.ipynb, accessed on 6 October 2026) using MMseqs2 [34]. The predicted HsdM protein structures were aligned using a custom Python script based on the Bio.PDB module of BioPython v.1.87. Structures were superimposed by least-squares fitting of corresponding Cα atoms, and residues with Cα deviations ≥ 2.0 Å were highlighted as locally divergent. For visualization of predicted PDB protein structures and amino acid substitutions, the Python 3 module py3Dmol-2.5.5 was used. The script, predicted PDB files, and an interactive HTML file presenting the alignment results are available at Zenodo: https://zenodo.org/records/23039958, accessed on 6 October 2026.

Computer tools from the SMRT Link software package v.25.2.0.266456 were used for calling methylated nucleotides in the PacBio-sequenced genomes. First, the original DNA reads in BAM format were aligned and sorted against the assembled genome sequences of the respective *S. enterica* strains using the pbmm2 aligner. The sorted read alignments in BAM format preserve the base-calling kinetics used by the ipdSummary program to identify the locations of methylated nucleotides, additionally providing information on local coverage and the statistical significance of methylation predictions. Methylation motifs were then predicted by the motifMaker program based on the ipdSummary statistical analysis. Visualization of the distribution of methylated motifs across chromosomes was performed using the SeqWord Motif Mapper v.3.2.6 (https://github.com/chrilef/BactEpiGenPro, accessed on 6 October 2026) [35], which filters methylated sites by coverage ≥ 10 and QVAL ≥ 20 (corresponding to *p* ≤ 0.01). This program also calculates z-score deviations between the expected and observed numbers of methylated sites in promoter, coding, and non-coding genomic regions and estimates the statistical significance of these deviations.

### 2.7. Use of Generative AI

Generative AI (OpenAI ChatGPT 5.6 Sol) was used only to check and improve English grammar and for debugging the in-house Python scripts.

## 3. Results

### 3.1. Bacterial Isolates Included in the Study

Of the 22 bacterial isolates included in the study, 20 were identified as *Salmonella enterica*, whereas two isolates were subsequently identified as *Escherichia coli*.

Four clinical isolates were obtained from patients hospitalized with clinical manifestations of acute gastroenteritis. The patients ranged in age from 46 to 67 years and included three women and one man.

All four patients presented with moderate gastrointestinal disease. The most common clinical manifestations included diarrhea, abdominal pain, nausea, vomiting, fever and/or chills, general weakness, signs of dehydration, and loss of appetite. The frequency of diarrheal episodes ranged from more than five to approximately twenty episodes per day in the most severe cases. Physical examination revealed tenderness in the epigastric and periumbilical regions in most patients.

Information about the collection strains was obtained from the Collection records. According to these records, none of the hospitalized patients reported recent travel outside Kazakhstan, which was consistent with probable local acquisition. No severe complications or generalized forms of infection were observed; however, the severity of diarrheal symptoms and signs of intoxication required hospitalization and rehydration therapy.

### 3.2. Cultural and Morphological Characteristics of the Isolates

Microscopic examination of Gram-stained smears showed that all 22 investigated bacterial isolates were Gram-negative rods with rounded ends. Growth characteristics and colony morphology were assessed on SS agar and meat-peptone agar (MPA). Colonies were predominantly circular, with regular margins and smooth surfaces. Colony diameters ranged from 2 to 6 mm depending on incubation time. No pronounced signs of culture dissociation were observed.

### 3.3. Biochemical and Serological Identification

The biochemical properties of the investigated isolates were determined using standard differential diagnostic media and test systems. Two isolates, 8S and 20S, were identified as *Escherichia coli* and were retained in the dataset for comparative genomic analysis. The remaining 20 isolates exhibited biochemical profiles characteristic of the genus *Salmonella*.

These 20 isolates fermented glucose, mannitol, maltose, dulcitol, sorbitol, arabinose, rhamnose, and xylose with acid and gas production, but did not ferment lactose or sucrose. They grew on Simmons citrate medium, produced hydrogen sulfide (H_2_S), and reduced nitrates to nitrites, while showing no indole production or gelatin hydrolysis.

All 20 *S. enterica* isolates yielded positive agglutination reactions with polyvalent O-antisera of groups A–E. The results of serotyping according to the White-Kauffmann-Le Minor scheme and multilocus sequence typing (MLST) are presented in Table 2.

### 3.4. Genome Sequencing and Phylogenetic Analysis

Whole-genome DNA sequences were obtained by sequencing the isolated samples on an Illumina sequencing platform. Six samples were additionally sequenced using the PacBio Revio platform to investigate the profiles of epigenetic modifications in genomic DNA.

To assemble DNA fragments generated by Illumina sequencing into contigs, the program SPAdes was used [26]. The MOB-suite software Version 3.1.9 [28] was employed to identify plasmid contigs. For complete genome assembly, the ILLAMINA pipeline (https://zenodo.org/records/15227656, accessed on 6 October 2026) was applied. This pipeline integrates de novo genome assembly results with reference-based assembly approaches. The genome assembly results are presented in Table 3.

**Figure 1:**
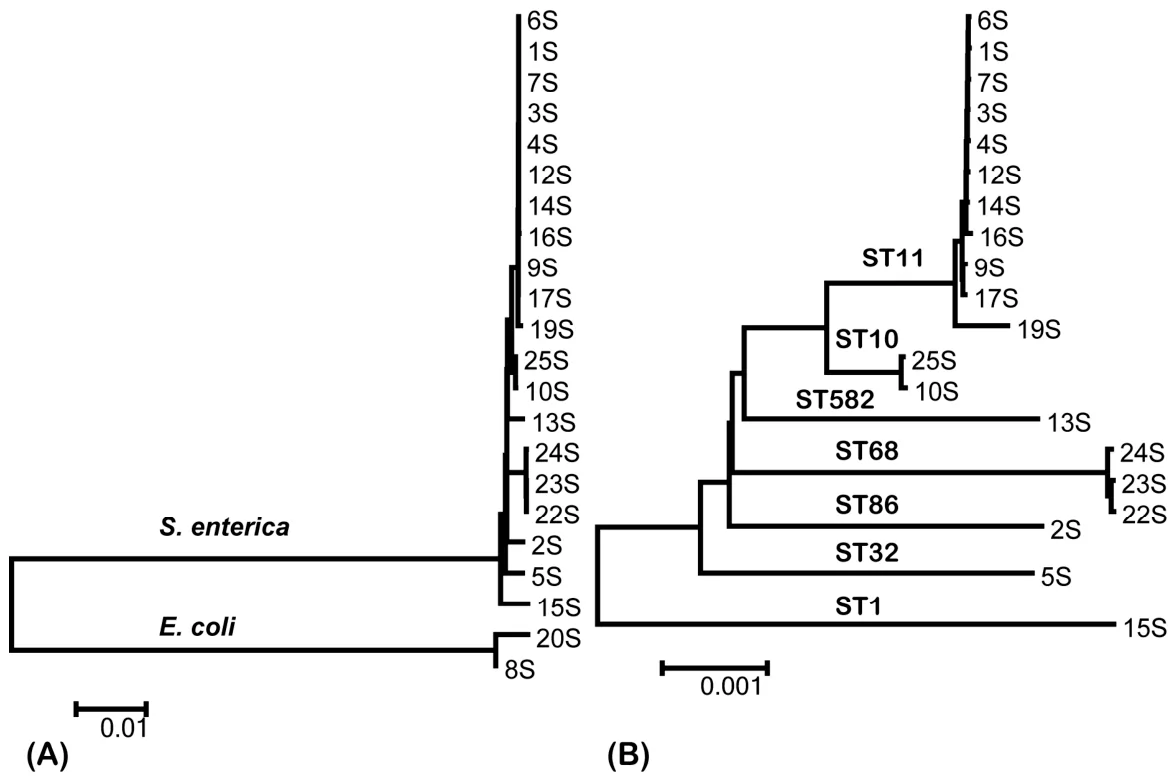
Phylogenetic relationships (**A**) between *S. enterica* and *E. coli* isolates and (**B**) among *S. enterica* isolates, with branches labeled according to MLST type.

To perform phylogenetic comparison, a matrix of single nucleotide polymorphisms (SNPs) in the core regions of all sequenced genomes, including two *E. coli* strains, was created using the Panaroo pangenome analysis tool [31]. In total, 1,960,174 SNPs with alternative allelic states in at least one genome were identified. To remove strain-unique SNPs, which could appear during laboratory cultivation of the collection strains, the matrix was filtered to retain SNPs with alternative allelic states confirmed in at least two genomes. The filtered matrix, containing 1,856,744 SNPs, was used for phylogenetic inference by the Neighbor-Joining algorithm implemented in MEGA12. The resulting phylogenetic tree confirmed clear separation of *S. enterica* isolates from *E. coli* strains (Figure 1A). For better resolution among *S. enterica* isolates, the *E. coli* strains were removed from the alignment. Phylogenetic grouping of the *Salmonella* strains was fully consistent with MLST (Figure 1B).

It was found that most of the selected *S. enterica* strains, with the exception of strain 16S, contained from one to four plasmids. A detailed characterization of the plasmid genomes is presented in Table 4.

### 3.5. Virulence and Antibiotic Resistance Gene Diversity

**Figure 2:**
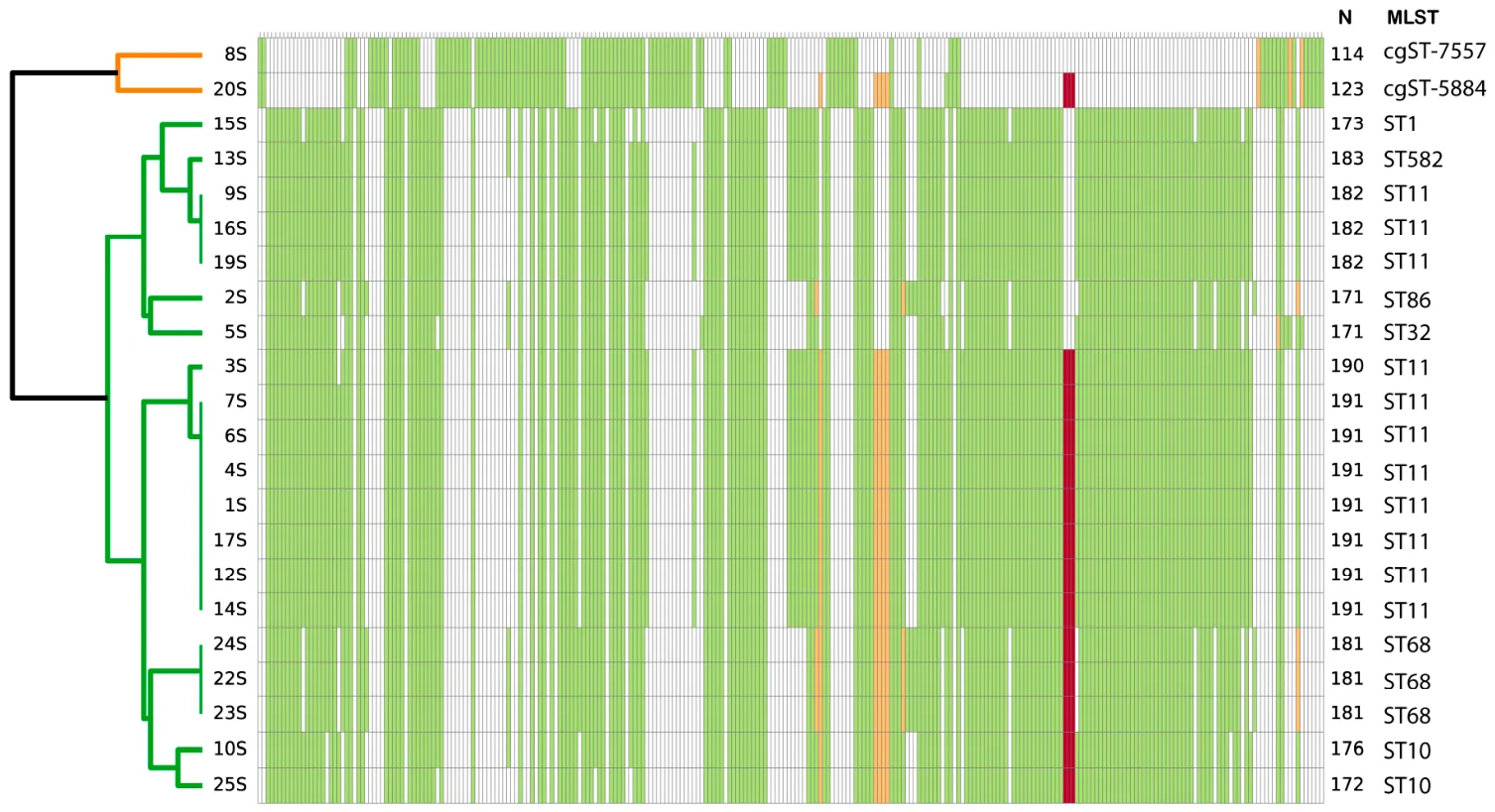
Presence-absence matrix of virulence genes in the genomes of the sequenced strains. White cells indicate absence of a given gene, while green cells indicate the presence of a single copy. Yellow and red cells denote the presence of two and three gene copies, respectively. Numbers on the right side of the plot indicate the total number of virulence genes identified in each genome. These numbers are followed by predicted MLST sequence types of the strains. Different colors of the tree branches indicate clusters of *S. enterica* (green) and *E. coli* (orange) strains.

The search for virulence genes was performed against the VFDB database using the Abricate program. Antibiotic resistance genes were identified using the RGI tool. Presence-absence matrices of virulence and resistance genes across different strains are shown in Figure 1 and Figure 2, respectively. Full lists of identified genes are given in respective Tables S1 and S2. Based on these matrices, cladograms representing similarity in gene patterns were constructed, and MLST sequence types identified for each strain are shown.

A substantial proportion of virulence genes comprised those encoding secretion systems responsible for delivering toxins and effectors into host cells, as well as genes encoding the effectors themselves. In particular, the type II secretion system (T2SS) was represented by the genes *gspC–gspM*; the type III secretion system (T3SS) by the operons *invA–invJ*, *spaO–spaS*, *prgH–prgK*, and *orgA–orgC*, along with their transcriptional regulators *hilA*, *hilC*, *hilD*, and *invF*, and genes encoding toxic effectors such as *sipA*, *sipB*, *sipC*, *sipD*, *sicA*, *sicP*, *iagB*, *iacP*, *sopA*, *sopB* (*sigD*), *sopD*, *sopD2*, *sopE2*, *avrA*, *sptP*, and *slrP*.

The type IV secretion system (T4SS) was primarily plasmid-encoded, although in some strains it was also duplicated by chromosomal genes. Some strains of the ST11 clade additionally contained genes associated with the type VI secretion system (T6SS).

Adhesin genes were also widely represented: *fimA–fimZ* (type 1 fimbriae); *lpfA–lpfE* (long polar fimbriae); *pefA–pefD* and *mrkA–mrkF* (other fimbrial systems commonly found in Enterobacteriaceae); *csgA–csgG* and *csgD* (curli biofilm proteins); as well as adhesins *bapA*, *fdeC*, *misL*, *shdA*, *pagN*, *rck*, *sinH*, *siiE*, and *yagV–yagZ*.

Iron acquisition and transport systems play a crucial role in microbial virulence, as iron ions are a limiting factor for bacterial growth during infection. The sequenced chromosomes contain genes involved in the synthesis of enterobactin (*entA–entF*, *entS*) and salmochelin (*iroB–iroN*), as well as genes encoding iron transport proteins (*fepA–fepG*, *fes*).

*Salmonella* strains produce and secrete systemic virulence factors such as *spvB*, *spvC*, and *spvD*, the cytolethal toxin *cdtB*, hemolysin *hly* and additionally the genes *pltA*, *pltB*, and *pltC* found in several strains.

To counteract host defense mechanisms, pathogenic bacteria rely on oxidative stress response genes such as *sodCI* and resistance to antimicrobial peptides produced by the host, mediated by the gene *ugd*. A number of genes are also involved in survival within macrophages, including magnesium ion transporters *mgtB* and *mgtC*, as well as macrophage survival genes *mig-14* and *mig-5*.

**Figure 3:**
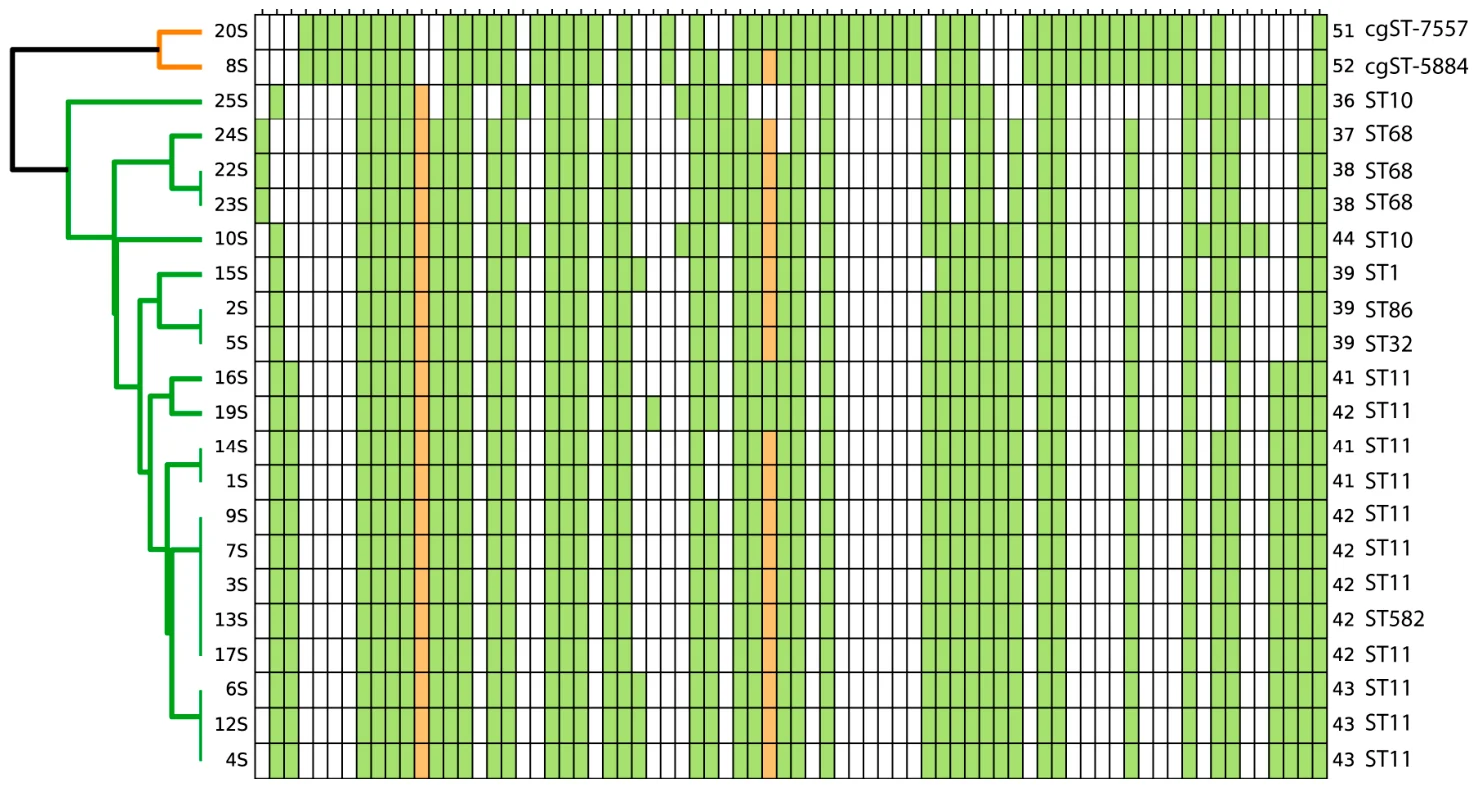
Presence-absence matrix of antibiotic resistance genes in the genomes of the sequenced strains. White cells indicate the absence of a given gene, while green cells indicate the presence of a single copy. Yellow cells denote the presence of two copies of the gene. Numbers on the right side of the plot indicate the total number of resistance genes identified in each genome. These numbers are followed by predicted MLST sequence types of the strains. Different colors of the tree branches indicate clusters of *S. enterica* (green) and *E. coli* (orange) strains.

Most of the antibiotic resistance genes shown in Figure 3 were located on chromosomes, with the exception of the *bla* TEM β-lactamase gene on plasmid AA474 in strain 19S. In contrast to plasmid-borne genes, the majority of chromosomal genes shown in Figure 3 represent typical components of the resistome of Enterobacteriaceae and are not specifically associated with resistance to clinically used antibiotics but rather include general resistance genes and regulatory elements controlling these genes.

From the perspective of acquired resistance to high doses of antibiotics, in addition to the plasmid-encoded TEM β-lactamase, important genes include *fosA7* (fosfomycin resistance), *sul1* (sulfonamide resistance), *tet*(*A*) and *tet*(*B*) (tetracycline resistance), and *aadA2*, APH(3′)-Ia, and APH(3′)-IIa (aminoglycoside resistance), although their presence in the genome alone does not necessarily indicate a broadly drug-resistant phenotype. Resistance-associated mutations were also detected (Table S2). In particular, well-known resistance-associated mutations were identified in the genes *gyrA* and *parC* (fluoroquinolone resistance), as well as *uhpT* (fosfomycin resistance).

The genomes of *E. coli* strains 8S and 20S were also enriched in virulence associated genes; however, the patterns of these genes differed from those observed in the *Salmonella* isolates.

### 3.6. Patterns of Nucleotide Methylation and Epigenetic Modifications

To verify methylation patterns, six strains (1S, 2S, 10S, 14S, 15S and 19S) were selected for additional sequencing on the PacBio Revio sequencing platform. The strains were selected to represent different serotypes and clades identified in the phylogenomic analysis (Figure 1B). To avoid possible effects of growth conditions on methylation patterns, the bacterial cultures were grown for DNA extraction at identical conditions explained in the Materials and Methods.

The genomes of all these strains were characterized by adenine methylation typical of Enterobacteriaceae on both DNA strands within the nucleotide sequence G*at*C (here and below, methylated nucleotides and the corresponding bases on the complementary strand are indicated in italicized lowercase letters). This methylation is controlled by the DAM methyltransferase (MTase) gene, which is not associated with restriction-modification (R-M) systems. Two copies of this gene were identified on the chromosomes of the sequenced *S. enterica* strains. In addition, an extra copy of the *dam* gene was detected on plasmid AF598.

The orphan DAM MTase, despite not being associated with an R-M system, is one of the most abundant MTases in enterobacteria that methylates genomic DNA at G*at*C motifs, reaching nearly 100% coverage of all available motifs at different growth conditions [35,36]. In all PacBio-sequenced genomes, methylation coverage at G*at*C motifs was above 99%. For example, in strain 1S, 37,677 of the 37,706 GATC motifs found in the genome were methylated. This consistently high level of DAM-specific methylation across strains may serve as a methodological positive control, ensuring high confidence in methylation calling.

**Figure 4:**
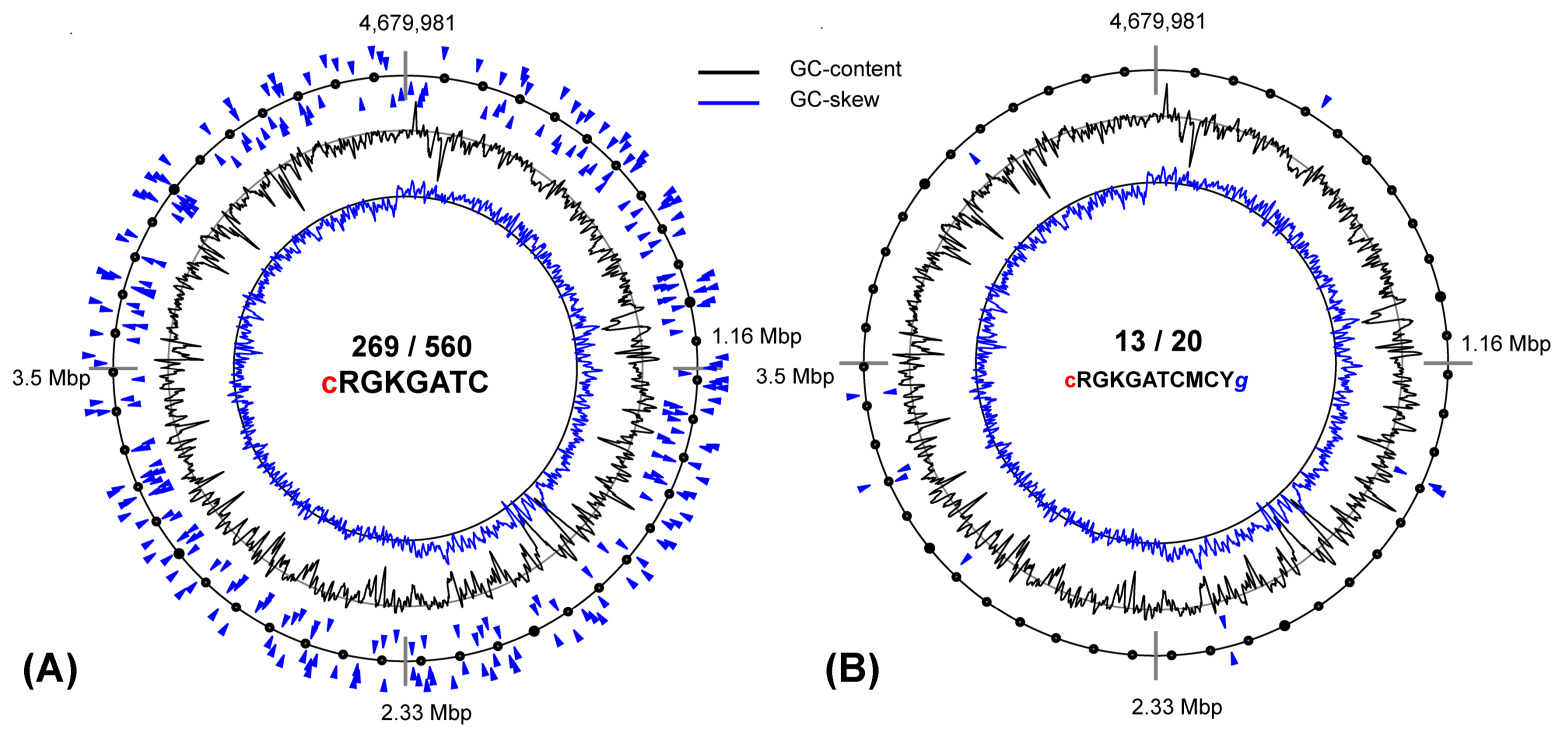
Patterns of cytosine methylation in strain 1S at (**A**) *c*RGKGATC and (**B**) *c*RGKG*at*CMCY*g* motifs. The positions of methylated sites along the circular chromosome are indicated by blue triangles. Local variations in GC skew (blue) and GC content (black) within 5000 bp sliding windows are represented by inner circular histograms.

DAM MTase is a dual-function enzyme that, in addition to adenine methylation, also catalyzes methylation of adjacent cytosine residues at motifs *c*RGKG*at*C, as well as at super-palindromic motifs *c*RGKG*at*CMCY*g* [35,36]. Analysis of the *S. enterica* genomes confirmed cytosine methylation in the same sequences associated with G*at*C motifs. Whereas adenine methylation at G*at*C motifs approached 100%, the associated cytosine methylation occurred in only approximately 50% of CRGKG*at*C and *c*RGKG*at*CMCY*g* motifs across different genomic regions and strains, suggesting a possible role for this methylation in the regulation of gene expression [35]. Cytosine methylation patterns at *c*RGKGATC and *c*RGKG*at*CMCY*g* motifs for strain 1S are shown, respectively, in Figure 4A and Figure 4B.

DCM is another orphan MTase common in Enterobacteria that methylates cytosine residues in C*c*W*g*G motifs [20,21]. Genes encoding DCM cytosine-5-methyltransferase were found in all sequenced genomes, and genome 5S contains two copies of this gene, all located on the chromosomes. Although cytosine methylation at CCWGG motifs was abundant, with around 7000 methylated sites identified in all genomes, this corresponded to only approximately 35% of the available methylation sites, leaving many cytosine residues within these motifs unmethylated.

An unexpected finding was the detection, in all genomes, of stable signals of epigenetic modifications involving cytosine in the semi-conservative motif *c*CAGGBRD and guanine in the motif GN*g*GTVVCRD. Although this cytosine methylation overlaps with the DCM-specific CCWGG motifs, the first cytosine in the motif is methylated, whereas DCM MTase methylates the second cytosine of this motif. The number of methylated *c*CWG*g* motifs was similar to the number of methylated C*c*W*g*G motifs, with around 7000 sites per genome; however, these two methylation patterns did not overlap, as no *cc*W*gg* methylated motifs were detected.

Epigenetic modifications of guanine are usually associated with oxidation to 8-oxoguanine; however, guanine oxidation typically occurs spontaneously and is not associated with specific sequence motifs. These guanine- and cytosine-associated epigenetic modifications were interrelated, since the motif CCAGG is complementary to the motif GGTVV. The GN*g*GTVVCRD sequences were found more frequently on the leading replichore of the chromosome, with a statistically significant correlation between motif frequency and GC-skew in sliding windows ranging from 0.10 to 0.17 (mean 0.13; *p* = 0.05). Modified guanine residues within these motifs were more frequent in coding regions (Z-score 2.36 ± 0.05, *p* = 0.018), while showing a random distribution across non-coding and promoter regions.

In contrast, the motif *c*CAGGBRD occurred more frequently on the lagging replichore, with correlation coefficients between the methylation frequency and GC-skew ranging from −0.16 to −0.08 (mean −0.12; *p*-value < 0.001). These motifs were more frequent in non-coding regions (Z-score 6.9 ± 0.03, *p*-value < 0.001) and less frequent in coding regions (Z-score −7.43 ± 0.04, *p*-value < 0.001).

While orphan DAM and DCM MTases are abundant, DNA methylation in bacteria is usually associated with the activity of MTases paired with restriction enzymes (REs), forming R-M complexes. Methylation of genomic DNA at specific nucleotide sequences (motifs) protects native DNA from RE activity, whereas invading DNA from bacteriophages or conjugative plasmids remains unprotected and is degraded by REs. Thus, R-M systems function as a form of “immunity” in bacterial cells against bacteriophages and conjugative plasmids [37].

The search for R-M operons in the sequenced genomes was performed using the DefenseFinder program [33] (Table 5).

Two R-M operons belonging to types I and III were identified in most genomes. Type I R-M systems were detected in all strains except 15S (Table 5). Type III R-M systems were absent in *E. coli* 8S and 20S. Instead, these two strains each contained two alternative type IV R-M operons. The largest diversity of R-M operons was observed in genome 25S, which contained R-M systems of types I, II (two operons), IIG-2, and III. It should be noted that the presence of R-M operons on the chromosome does not guarantee their activity in genome methylation or phage defense. The methylation patterns were verified in six strains sequenced on the PacBio Revio sequencing platform.

Intensive adenine methylation at CAG*a*G motifs was detected in all sequenced genomes and most likely is associated with the type III R-M system, the genes of which were also identified in all six sequenced strains. The methylated motifs were predominantly located in non-coding regions of the genome (Z-score 8.98 ± 0.02, *p*-value < 0.001) and in promoter regions of genes (Z-score 2.31 ± 0.08, *p* = 0.021) but were rarely found within coding regions of the genome (Z-score −10.59 ± 0.02, *p* = 0.001).

**Figure 5:**
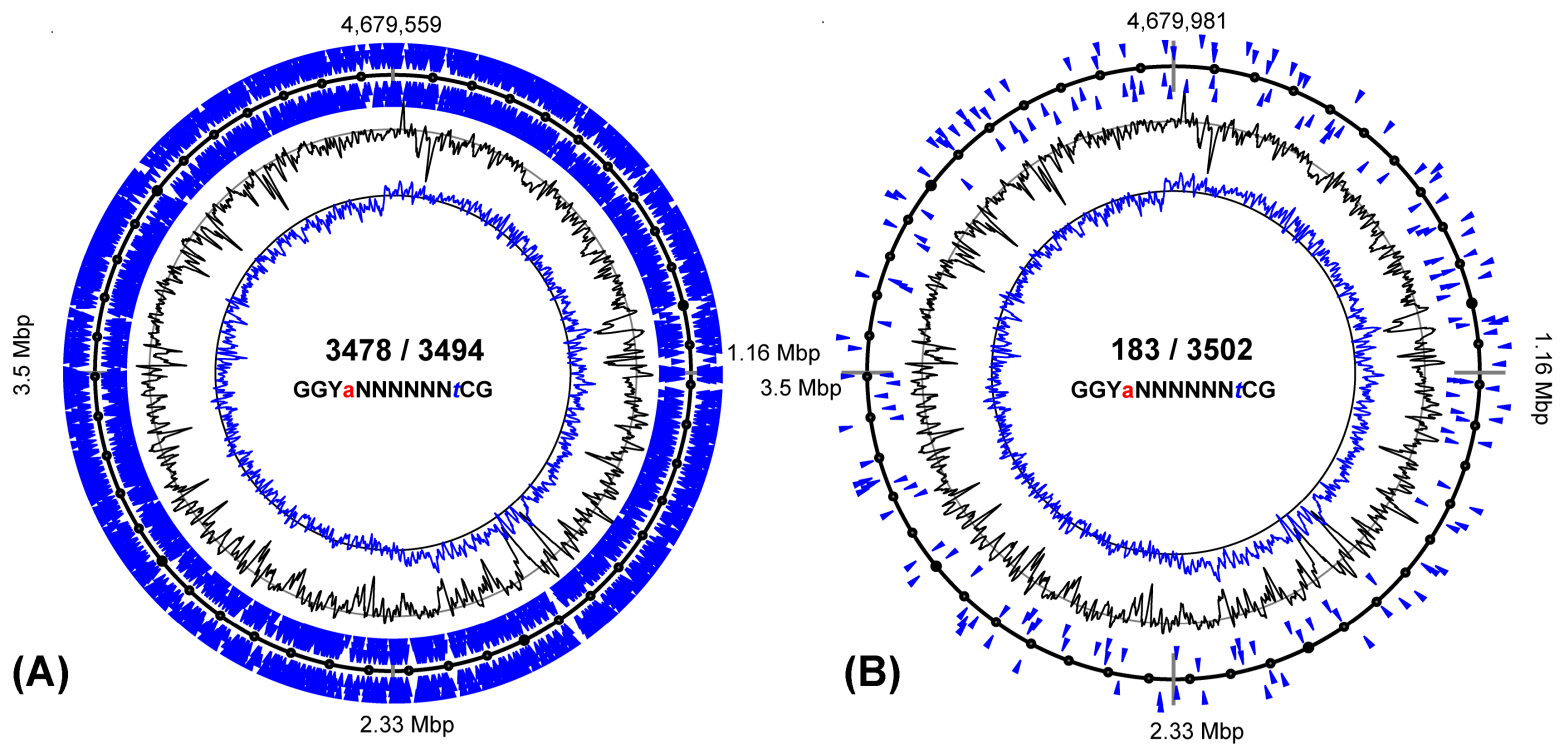
Patterns of adenine methylation at GGY*a*NNNNNN*t*CG motifs in (**A**) 19S and (**B**) 1S strains. The positions of methylated sites along the circular chromosomes are indicated by blue triangles. Local variations in GC skew (blue) and GC content (black) within 5000 bp sliding windows are represented by inner circular histograms.

Genes encoding type I R-M systems were identified in five of the six PacBio sequenced strains; strain 15S lacked a type I R-M system according to Table 5. However, analysis of epigenetic modification patterns revealed type I specific adenine methylation on both DNA strands at the motif GGY*a*NNNNNN*t*CG only in strain 19S (Figure 5A). In the other five strains, no more than 5% of GGYANNNNNNTCG motifs were methylated, as illustrated for strain 1S in Figure 5B, which can be considered as a residual or background methylation. In strain 19S, this methylation predominantly targeted coding sequences (Z-score 3.24 ± 0.03, *p* = 0.001) and was rare in promoter regions (Z-score −6.36 ± 0.1, *p*-value < 0.001), while showing a random distribution in non-coding regions.

## 4. Discussion

An important feature of this study is its retrospective design based on an archived bacterial collection rather than prospective epidemiological sampling. The investigated strains originated from different time periods and independent clinical or veterinary episodes and were selected to characterize the range of genetic and epigenetic diversity preserved within the collection. Direct phenotypic comparisons between historical and recent isolates are limited because some strains had been maintained under laboratory conditions for many years and the original clinical or veterinary records were incomplete. The genomic data nevertheless provide information on the diversity of lineages and accessory genetic determinants preserved in the collection and establish a molecular framework for comparison with contemporary clinical, veterinary, and environmental isolates. Strain-unique SNPs, which could appear during the long-term cultivation at laboratory conditions, were removed from consideration.

### 4.1. Genotyping and Genotype Distribution

This study confirms previous reports that ST11 is a frequent sequence type among Asian *S. enterica* isolates associated with food poisoning [18,38,39].

A large number of plasmids shown in Table 3 and Table 4 indicate their significant role in the biology and survival strategy of *S. enterica*. Most of the isolated cultures contained two plasmids: AF598 and AB232. Plasmid AF598 carries several genes and operons important for bacterial metabolism. For instance, the operon comprising the genes *rhaT*, *rhaR*, *rhaS*, *rhaA*, *rhaB*, *rhaD*, and *rhaM* provides a complete pathway for L-rhamnose utilization. L-rhamnose metabolism is common among bacteria inhabiting plant-associated environments, where rhamnose-containing polysaccharides are abundant [40]. This suggests that this plasmid may play an important role in the long-term survival of the pathogen in the external environment, particularly in the rhizosphere [41]. Stress response genes present on this plasmid, such as *cpxA*, *cpxR*, *cpxP*, and *sodA*, are typically not directly associated with virulence but may also contribute to environmental persistence. The usage of metabolic, stress-response, and some classical virulence functions by *Salmonella* during plant colonization was reported in other publications [42,43,44].

In contrast, another large plasmid, AB232, present in most of the sequenced strains, is clearly associated with pathogen virulence. This plasmid carries a complete set of genes encoding a type IV secretion system (T4SS), including *virD4*, *virB4*, *virB9*, *virB11*, and *ptlE*. These genes increased bacterial uptake and survival in epithelial cells and macrophages [45]. In addition, the genes *spvB* and *spvC* encode virulence factors responsible for the destruction of host cell cytoskeletal microfilaments [46,47] and suppression of the immune response and intracellular survival [48]. Other virulence-associated factors detected in this plasmid include the genes *rfaH*, *vsdE*, *mkaB*, *mkaC*, and *invA* mediating LPS biosynthesis [49], host cell invasion [50] and other virulence gene activation [51]. Furthermore, the plasmid contains conjugation genes and numerous transposons and integrases, facilitating plasmid transfer between cells and the integration of genetic material into the core genome of the pathogen. The absence of plasmid AB232 may indicate reduced virulence; however, in strain 19S this plasmid was replaced by a smaller plasmid carrying a similar repertoire of virulence genes, thereby maintaining pathogenicity of the strain while potentially increasing growth rate.

Other plasmids were specific to individual strains. For example, strain 19S, which lacked the two aforementioned plasmids, harbors a large plasmid AA474, in which the *bla* gene encoding TEM β-lactamase was identified. A BLASTN search showed that this plasmid has been identified predominantly in pathogenic *E. coli* and, less frequently, in *S. enterica* strains. The plasmid also carries the *cia* gene encoding a colicin Ia immunity protein, which may protect the bacterial cell against the corresponding colicin and contribute to competitive interactions with other Enterobacteriaceae [52]. Other genes on this plasmid are involved in conjugation and horizontal gene transfer. The same strain also carries another medium-sized conjugative plasmid, AA619, which is also present in strains 10S and 25S. This plasmid contains the genes *virB4*, *virB8*, *virB9*, and *virB11* of the type IV secretion system (T4SS), which is involved in pathogenesis. In strains 10S and 25S, these genes duplicate the T4SS genes found on the large plasmid AB232.

Strain 19S also contains a small plasmid, AA974, which is also found in strain 13S. This plasmid carries four genes of unknown function. Two other small plasmids, AD529 and AB241 (Table 4), likewise do not contain functional genes except for the regulatory gene *rop*, which is involved in the replication of ColE1-type plasmids [53].

Clustering of strains based on virulence and antibiotic resistance gene patterns shown in Figure 1 and Figure 2 demonstrated that strains belonging to the same genotype (MLST ST) and serotype (Table 2) are not always homogeneous with respect to the presence or absence of genetic factors associated with horizontal gene transfer. Distinct combinations of virulence and resistance genes arise within bacterial clades as a result of horizontal gene transfer, and natural selection in specific ecological niches, as well as differences in microbial survival strategies and predominant routes of transmission [54,55]. In general, representatives of the less common genotypes (ST1, ST582, ST86, ST32, ST68, and ST10) in our collection were characterized by a reduced repertoire of virulence factors compared with ST11 isolates. A comparatively diverse repertoire of virulence determinants has also been reported for ST11 *S. enterica* Enteritidis isolates in other studies, including plasmid-associated *spv*, *rck*, and *pef* genes and determinants associated with several *Salmonella* pathogenicity islands [39,56,57,58]. However, virulence-gene repertoires vary considerably among *S. enterica* lineages and individual isolates, and the presence of these determinants should not itself be interpreted as evidence of increased phenotypic virulence. It is also worth mentioning that the presence of virulence and AMR determinants in the studied genomes without experimental validation should not be interpreted as evidence of the corresponding virulence or antimicrobial resistance phenotype.

### 4.2. Epigenetic Profiling

In recent years, an increasing number of studies have suggested that changes in genome methylation patterns may directly or indirectly influence pathogen virulence and antimicrobial resistance [20,59,60,61,62]. MTase genes and entire R-M operons are transferred between bacteria through horizontal gene exchange mediated by plasmids or bacteriophages [63]. Such exchange not only provides bacteria with new defense mechanisms against phages but also affects patterns of gene expression and regulation through the emergence of new methylated nucleotide sites within genes and promoter regions [64].

Acquired R-M operons gradually degenerate as a result of mutations. Consequently, bacterial genomes often contain more individual MTases and R-M operons than detectable DNA methylation motifs [36]. The degradation of R-M systems usually begins with an inactivation of RE, causing MTases to lose tight control over their activity and specificity. Degradation of R-M systems makes bacterial strains more vulnerable to phage attacks but at the same time increases the likelihood of gene exchange via plasmids and transposons [36]. Invasive strains are relatively well protected from phages due to their intracellular parasitic lifestyle, whereas the acquisition and exchange of virulence factors and antibiotic resistance genes become critically important for their evolutionary success.

Because *Salmonella* is characterized by a dual lifestyle involving host invasion and hospital-associated infections, as well as persistence in water and plant rhizospheres populated by highly competitive microorganisms and bacteriophages [65,66,67], these bacteria require different survival strategies across highly diverse ecosystems. This may explain the presence of the two plasmids, AB232 and AF598, in the majority of the sequenced strains, as they provide genes required either for virulence or for environmental survival. It can be hypothesized that R-M systems may also be fine-tuned for adaptation to different environments, while R-M-associated and orphan MTases may participate in the regulation of gene expression [21].

The unexpected lack of DNA methylation activity at type I motifs in the sequenced genomes, despite the abundance and high level of conservation of the type I R-M operon, may represent an example of such tightly regulated R-M systems, which so far have been poorly characterized in the literature.

The Type I R-M gene cluster comprises three genes: the *hsdR* type I R-M endonuclease; the *hsdM* N6-DNA MTase; and the *hsdS* motif-recognition specificity subunit. In most *S. enterica* strains analyzed in this study, the R-M-associated gene *mrr* is located next to this cluster on the opposite DNA strand, and this arrangement of two R-M systems was reported before in other sequenced *Salmonella* and *Escherichia* strains [68,69,70]. Gene *mrr* encodes a Type IV endonuclease that cleaves methylated DNA [69,71]. If active, these genes together may protect the host cell against two types of incoming foreign DNA: DNA lacking the methylation pattern established by the Type I methyltransferase and DNA carrying methylation introduced by methyltransferases absent from the host. However, the DefenseFinder program did not identify this gene as an active Type IV R-M system (Table 5). Thus, this gene may play another role in the conserved *mrr*|*hsdR*–*hsdM*–*hsdS* gene arrangements characteristic of many *S. enterica* isolates. 

**Figure 6:**
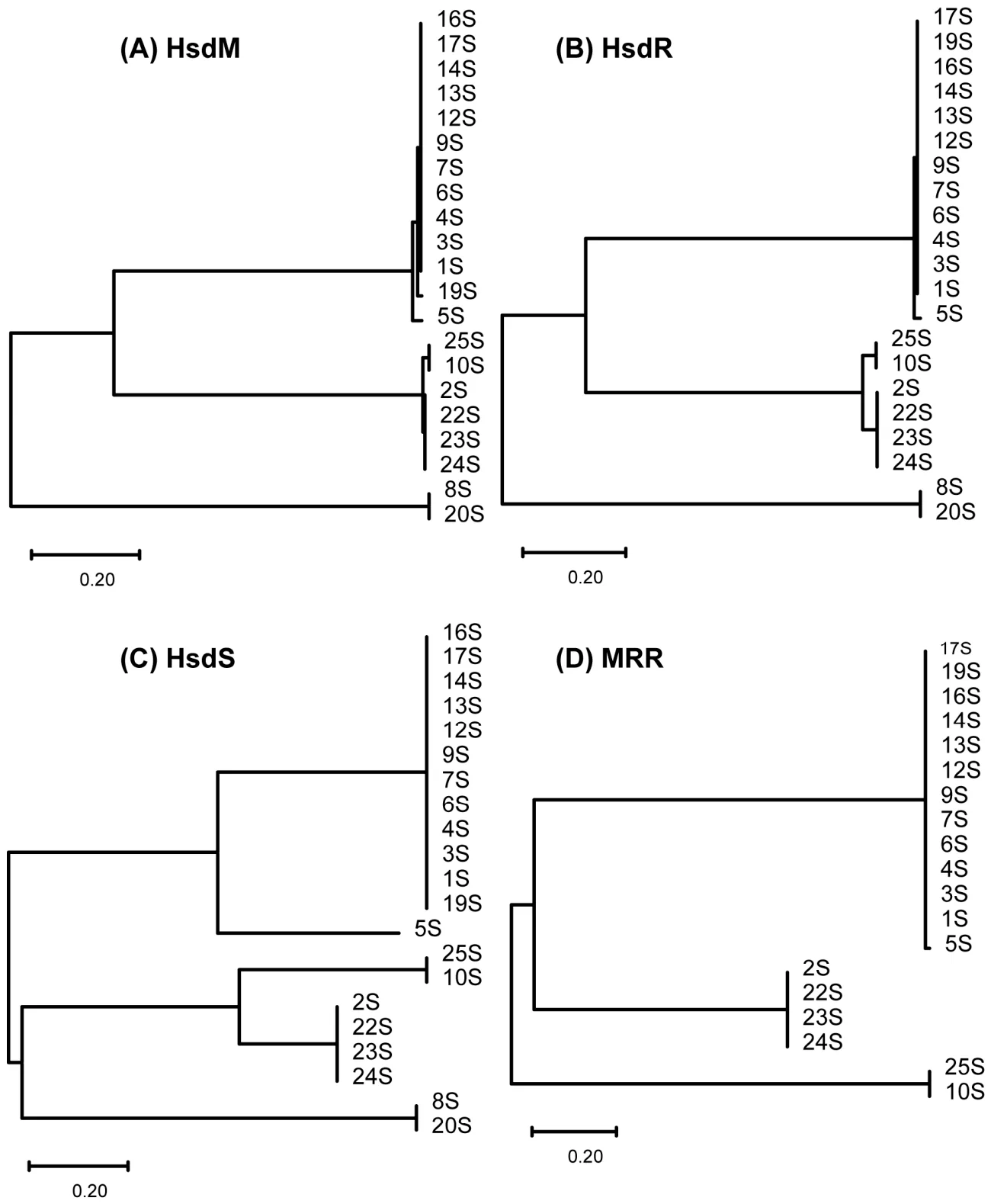
Neighbor-joining cladograms showing the sequence similarity of proteins encoded by type I R-M operons: (**A**) HsdM; (**B**) HsdR; (**C**) HsdS; and (**D**) MRR.

The *mrr* gene was not found next to the Type I R-M operon in the sequenced genomes of *E. coli* strains 8S and 20S. Another distinctive feature of these strains was the insertion of the ATP-dependent RNA helicase gene *srmB* and the DNA-processing protein gene *dprA* within the Type I R-M operon. Gene-by-gene comparisons (Figure 6A–D) also suggested that the Type I R-M operons in different *S. enterica* and *E. coli* strains may have different evolutionary origins. The *hsdS* gene, encoding the motif-recognition specificity subunit, was the most variable between groups, making it potentially a useful marker for distinguishing *S. enterica* lineages. Within ST groups, however, the operons were highly conserved: no nucleotide substitutions were detected in either coding or intergenic regions.

An exception was observed in strain 19S, which differs from the other strains of the ST11 genotype by multiple nucleotide substitutions in *hsdM*, resulting in seven amino acid substitutions within two variable regions: (L103I, E105A, Y109N, A112S, and N119A) and (T363A and H366N). In this notation, the first letter represents the amino acid state in the majority of ST11 strains, the last letter represents the amino acid state in strain 19S, and the number indicates the codon position.

**Figure 7:**
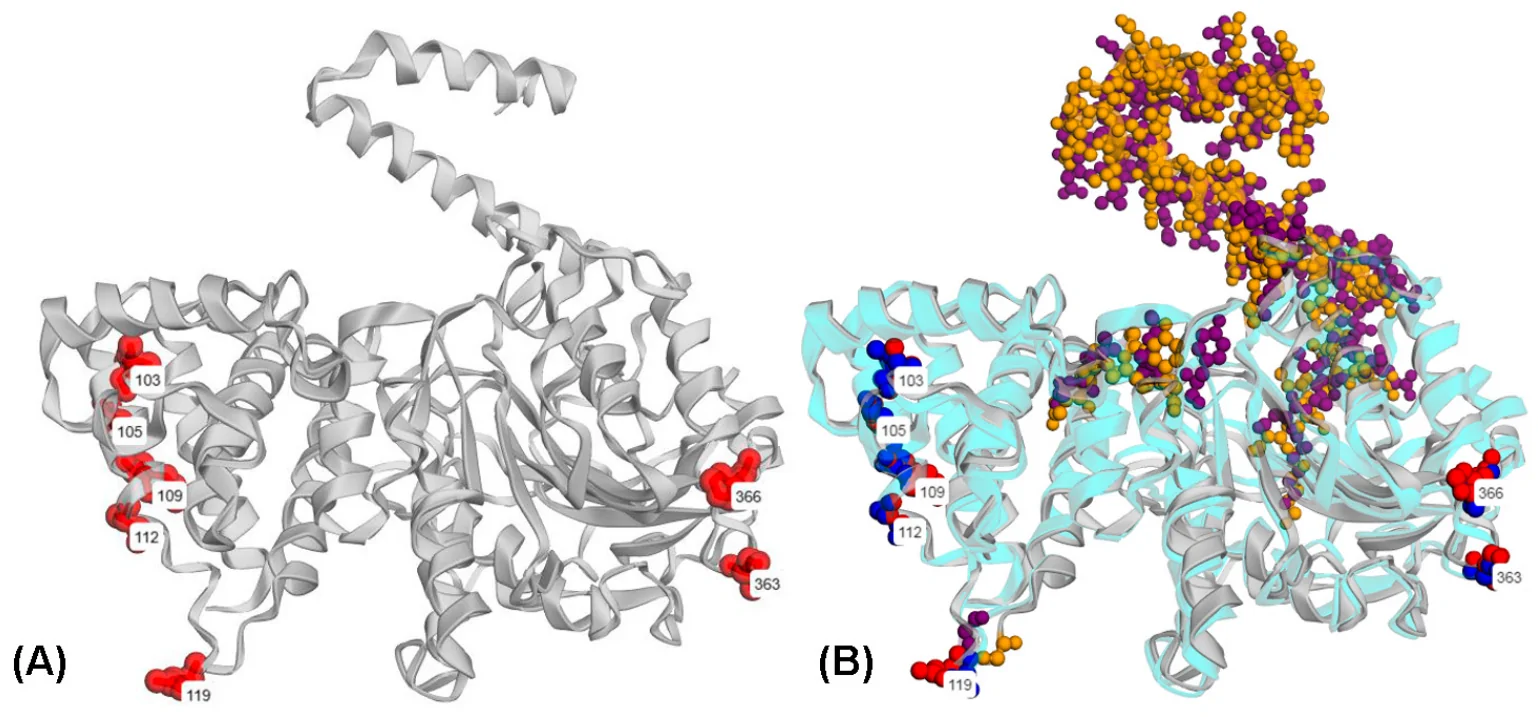
Mapping of amino acid substitutions onto predicted three-dimensional structures of HsdM. (**A**) Predicted structure of HsdM from strain 19S, shown as a gray ribbon. Substituted residues are highlighted in red and labeled by amino acid position. (**B**) Structural alignment of HsdM from strain 19S (query; light blue ribbon) with HsdM from strain 1S (reference; gray ribbon). Substituted residues are shown in ball-and-stick representation and labeled by position. Regions with structural deviations greater than 2 Å are highlighted in purple for the query and orange for the reference.

Surprisingly, mutant strain 19S was the only strain with detectable adenine methylation at Type I recognition motifs. Mapping the amino acid substitutions onto the predicted three-dimensional structure of the HsdM protein showed that they lie on the protein surface away from the C-terminal α-helical region implicated in HsdS binding (Figure 7A). Structural alignment of HsdM from strains 19S and 1S showed no obvious structural changes near the substituted residues (Figure 7B). Differences elsewhere, including in the putative HsdS-binding region, may reflect flexibility in the predicted structures; their functional significance remains uncertain. Further investigation is required to determine the possible role of these domains in the regulation of MTase activity, which could explain the active chromosomal methylation observed in the mutant strain as a result of escape from MTase suppression observed in other strains.

Figure 7B is a snapshot of an interactive HTML visualization deposited in Zenodo (https://zenodo.org/records/23039958, accessed on 6 October 2026) together with the corresponding PDB files and the Python code used for visualization.

Activation in MTase activity, in turn, leads to a redistribution of methylated DNA regions. For example, in the genome of strain 19S, which exhibited increased Type I-associated methylation activity, important genes such as the RNA polymerase gene *rpoB*, the methionine synthase gene *metH*, and the transmembrane transporter gene *xylP* became hypermethylated, each containing five methylation sites. In the other genomes, these genes remained unmethylated. Likewise, the promoter regions upstream of the *clpX* protease gene and the glutathione permease gene *gsiC* also remained unmethylated in those strains but methylated in 19S.

Hospital strain 19S, isolated in 2025, represents a distinct ST11 isolate within this dataset (Figure 1). This strain showed a distinct plasmid repertoire (Table 4) by replacing the large AF598 and AB232 plasmids, which are present in several other ST11 strains, with shorter plasmids enriched in virulence determinants and the *bla* TEM β-lactamase. The alteration in genome methylation coverage at Type I motifs GGY*a*NNNNNN*t*CG, from 0.995% in 19S to 0.05% in other PacBio-sequenced strains, is striking and may be associated with mutations in the HsdM MTase protein found in strain 19S. However, the association between these amino acid substitutions and the strong methylation phenotype requires further functional validation. Notably, all other genes, including the intergenic spacer regions within the *mrr*|*hsdR*–*hsdM*–*hsdS* complex remained intact (Figure 6). This implies the functional importance of this preserved Type I R-M system, raising the possibility that its activity may depend on environmental or physiological conditions.

HsdM plays a central role in Type I R-M activity. DNA methylation is carried out by a complex comprising one HsdS and two HsdM subunits, whereas restriction activity requires a complex containing one HsdS, two HsdM, and two HsdR subunits [72]. Thus, inactivation of HsdM abolishes both activities and prevents the cognate restriction enzyme from cleaving the host chromosome. We hypothesize that Type I R-M activity in clinical *Salmonella* isolates may be activated primarily upon exposure to phage-rich environments, whereas the system may be constitutively active in strain 19S because of its greater dependence on survival in open environments. This hypothesis requires experimental confirmation in future studies.

DAM-mediated methylation at GATC motifs was most stable and approached 99% of all detected motifs. In the current study, notable differences were observed in the GATC methylation of promoter regions upstream of the *prfB-2* and *yqcC* genes, where methylation was absent in strains 2S and 10S, but three methylation sites were present in the other four strains. Experiments with a DAM-deficient *S. enterica* serovar Typhimurium strain using an epithelial-cell invasion model showed strong attenuation of the mutant strain compared with the wild type [62]. However, it remained unclear whether this attenuation was associated with altered methylation at specific regulatory regions or resulted, at least in part, from the important housekeeping roles of DAM methylation in DNA replication, mismatch repair, chromosome segregation, and genome stability [73].

Previous studies have indicated that methylation within promoter regions can influence gene expression [36,60,63]. However, these publications also show that general associations between alternative methylation patterns and gene regulation have often been inferred using statistical approaches, while experimental studies involving MTase inactivation in different microorganisms have demonstrated effects that depend on the individual strain and experimental conditions. Here, we report the observed variations in the distribution of R-M-associated methylated sites; however, possible associations of these variations with *S. enterica* virulence, lifestyle adaptation, gene expression, and other phenotypic properties require experimental validation in future studies. The analysis of methylated sites not associated with the identified R-M systems further demonstrates the diversity of methylation patterns among the studied strains, without implying a specific functional role.

In contrast to the DAM methylation at adenine residues, DCM-mediated cytosine methylation at C*c*W*g*G motifs was partial, reaching approximately 35% of the available motifs. Variations in non-R-M methylation of *S. enterica* Typhimurium genomes during different growth stages were reported [21]. Another study showed that DCM-mediated methylation at CCWGG motifs in *Yersinia pestis* is temperature-dependent and may be involved in gene regulation during the transition from vector insects to warm-blooded hosts [74]. Similar numbers of methylated *c*CWG*g* sites were detected in each genome; however, these two methylation patterns did not overlap. Significant differences in frequencies of C*c*W*g*G and cCWG*g* methylation, ranging from no methylation to 10–13 methylated sites per gene body, were observed across the sequenced genomes in the *rpoC* DNA-directed RNA polymerase beta-subunit, *acrB_2* multidrug efflux pump, and several conserved hypothetical genes. Differential methylation at these motifs was also observed in the promoter regions of the following genes: *pdeN_2*; cyclic di-GMP phosphodiesterase, *lolA* outer-membrane lipoprotein carrier protein, *lldD* L-lactate dehydrogenase, *yecS_3* L-cystine permease, *yejB* inner membrane ABC permease, and in *dcuR/dcuS* transcriptional regulators.

It is plausible that the same DCM MTase may be responsible for both methylation patterns, with its substrate specificity influenced by the surrounding nucleotide context. For example, cCAGGBRD methylation was most frequently observed when the CCAGG motif was followed by a BRD sequence. However, the detection of epigenetic modification signals involving cytosine and guanine within the tandem repeats *c*CAGGBRD and GN*g*GTVVCRD suggests that epigenetic modifications associated with CCWGG motifs may arise from an overlap of several independent processes.

Tandem repeats of semi-conservative motifs unevenly distributed between bacterial chromosome replichores have previously been described as architecture-imparting sequences (AIMS), which serve as binding sites for proteins involved in DNA replication and repair processes, such as FtsK [75]. Earlier studies have shown that AIMS repeats can be methylated in some bacteria [35]. It can therefore be hypothesized that interactions with replication and repair proteins in *S. enterica* may induce structural changes in DNA that are recognized by the PacBio sequencing platform as epigenetic modifications. Previously, similar methylation of AIMS repeats was observed in *Staphylococcus aureus* [64]. The functional impact of these changes on genome activity requires further investigation.

### 4.3. Limitations

This retrospective analysis demonstrates substantial genetic and epigenetic diversity among *S. enterica* strains preserved in an archived collection and illustrates the information that can be recovered from historical bacterial resources using modern sequencing technologies. The observed genotype frequencies characterize the analyzed collection and should not be interpreted as estimates of population prevalence. Likewise, genomic detection of virulence- and AMR-associated determinants indicates genetic potential but does not by itself establish the corresponding phenotype. Despite these limitations, genomic characterization of archived strains provides a reference for comparison with newly emerging isolates and may support future epidemiological surveillance by identifying changes in circulating lineages, plasmid repertoires, resistance determinants, and other genomic characteristics over time.

The epigenetic analysis also has several limitations. DNA methylation was investigated in only six selected strains, and biological replicates of PacBio sequencing were not performed. Although cultures used for DNA extraction were grown under standardized conditions, methylation patterns may depend on growth phase and environmental conditions. Therefore, the observed differences between strains should not be interpreted as stable strain-specific characteristics under all growth conditions.

The functional consequences of the detected methylation patterns were not experimentally investigated. Enrichment or depletion of methylated sites in promoter, coding, or non-coding regions indicates non-random genomic distribution but does not demonstrate an effect on transcription. Similarly, associations between particular methylation patterns and virulence, antimicrobial resistance, environmental adaptation, or other phenotypic properties remain hypotheses that require experimental validation.

Finally, the relatively small and heterogeneous set of archived isolates selected for this study limits statistical comparisons between strains of human and animal origin, geographical regions, collection periods, and individual genetic lineages. These limitations do not affect the genomic characterization of individual isolates but restrict broader epidemiological generalization of the observed patterns.

## 5. Conclusions

*S. enterica* remains a frequent causative agent of acute intestinal infections, often with a tendency toward systemic multiple organ infection [1,8,9,10,11]. It is important to be able to identify sources of disease outbreaks in a timely manner and to accurately assess the epidemiological risks associated with disease spread. MLST genotyping enables the identification of genetic variants (clades and lineages) of pathogens belonging to the same species. However, it should be taken into account that MLST genotyping is based on the analysis of neutral mutations in core genes. These mutations usually do not carry direct biological significance and primarily serve as markers of common ancestry. Additional information on strain virulence and pathogenicity can be obtained by analyzing combinations of accessory genes, including virulence factors and antibiotic resistance genes.

In the present study, important differences were identified in the repertoires of virulence and antimicrobial resistance genes among *S. enterica* isolates from clinical and veterinary sources, predominantly originating from Kazakhstan. ST11 was the most represented sequence type among the selected strains; however, ST11 isolates showed variation in plasmid content and virulence- and resistance-associated gene profiles.

The frequent presence of plasmids AF598 and AB232 suggests that these plasmids may contribute to metabolic, stress-response, and virulence-associated functions, although their effects on environmental persistence or invasiveness were not experimentally tested. The recent clinical isolate 19S was notable for a distinct plasmid repertoire enriched in virulence- and antibiotic-resistance determinants.

Additional insights into the genetic variability of bacterial cultures can be obtained using modern sequencing technologies such as PacBio SMRT sequencing technology, which allows profiling of epigenetic modifications in microbial genomes.

The detection of MTase genes on bacterial chromosomes does not guarantee their functional activity, whereas DNA methylation profiles provide evidence of methylation activity associated with specific MTases and R-M systems. The biological consequences of differences in R-M activity for environmental persistence, invasiveness, and horizontal gene transfer require further experimental validation. Furthermore, differences in the repertoire and activity of R-M systems and individual MTases enable more precise discrimination between bacterial clades within the same genotype through differences in methylation profiles. The influence of methylation and other types of nucleotide epigenetic modifications on gene expression in *S. enterica* remains an important subject for future investigation using transcriptomic approaches. The *S. enterica* strains sequenced in this project may serve as optimal model organisms for future studies on the role of DNA methylation and other epigenetic nucleotide modifications in the regulation of gene expression in pathogens, particularly strain 19S, which exhibited increased DNA methylation at type I motifs affecting numerous genes and promoter regions.

Thus, the ultimate value of the genome-wide and epigenetic characterization of the investigated strains lies in creating a foundational genetic platform for the comparative genomics of *S. enterica* in Kazakhstan. These results contribute to public health and epidemiology by providing reference data to reconstruct evolutionary trends of the *S. enterica* population, while the identification of strain 19S provides a solid basis for future functional and transcriptomic studies regarding the effect of genome methylation on bacterial adaptation.

## Figures and Tables

**Table 1:** Isolation origins, dates of deposit in the National Collection of Microorganisms (NCM), and geographic origins of the bacterial strains used in this study.

Strain	Isolation Origin	Date of Deposit in NCM	Region
1S	Human	26 February 1977	Almaty city
2S	Animal	2 July 1977	Almaty region
3S	Animal	26 June 2007	Almaty region
4S	Rabbit (*Oryctolagus cuniculus*)	15 March 1989	Almaty region
5S	Animal	15 March 1989	Almaty region
6S	Rabbit (*O. cuniculus*)	15 March 1989	Almaty region
7S	Wood mouse (*Apodemus sylvaticus*)	25 April 1963	Kyrgyzstan
8S	Animal	25 April 1963	Kyrgyzstan
9S	Animal	2 February 1957	Almaty region
10S	Animal	25 November 2009	Almaty region
12S	Animal	25 November 2009	Turkestan region
13S	Animal	25 November 2009	Turkestan region
14S	Animal	25 November 2009	Shymkent city
15S	Animal	25 November 2009	Shymkent city
16S	Human	18 September 2025	Almaty city
17S	Human	7 December 2025	Almaty city
19S	Human	18 August 2025	Almaty city
20S	Human	7 November 2025	Almaty city
22S	Ewe (abortion case)	10 November 2023	Zhambyl district, Almaty region
23S	Aborted sheep fetus	4 January 2024	Osakarovsky district, Karaganda region
24S	Horse (*Equus ferus caballus*)	27 February 2012	Talgar district, Almaty region
25S	Calf (*Bos taurus*)	9 April 2013	Talgar district, Almaty region

**Table 2:** Antigenic formulas, MLST sequence types, and serovar assignments of the analyzed bacterial isolates.

Strain	Serotype *	MLST	Serovar
1S	9:g,m:-	ST11	*S. enterica* Enteritidis
2S	4:b:1,2	ST86	*S. enterica* Paratyphi B
3S	9:g,m:-	ST11	*S. enterica* Enteritidis
4S	9:g,m:-	ST11	*S. enterica* Enteritidis
5S	7:r:1,5	ST32	*S. enterica* Infantis
6S	9:g,m:-	ST11	*S. enterica* Enteritidis
7S	9:g,m:-	ST11	*S. enterica* Enteritidis
8S	Unidentified	EC ST2	*E. coli*
9S	9:g,m:-	ST11	*S. enterica* Enteritidis
10S	9:g,p:-	ST10	*S. enterica* Dublin
12S	9:g,m:-	ST11	*S. enterica* Enteritidis
13S	8:e,h:1,5	ST582	*S. enterica* Kottbus
14S	9:g,m:-	ST11	*S. enterica* Enteritidis
15S	9:d:-	ST1	*S. enterica* Typhi
16S	Unidentified	ST11	*S. enterica* Enteritidis
17S	9:g,m:-	ST11	*S. enterica* Enteritidis
19S	Unidentified	ST11	*S. enterica* Enteritidis
20S	Unidentified	EC ST2	*E. coli*
22S	7:c:1,5	ST68	*S. enterica* Choleraesuis
23S	7:c:1,5	ST68	*S. enterica* Choleraesuis
24S	7:c:1,5	ST68	*S. enterica* Choleraesuis
25S	9:g,p:-	ST10	*S. enterica* Dublin

* Antigenic formulas are given according to the White-Kauffmann-Le Minor serotyping scheme [25]. Serovar entries were rechecked against the original serotyping records.

**Table 3:** Genome assembly characteristics of the analyzed bacterial isolates.

Strain	Chromosome Length (bp)	Chromosome GC-Content (%)	Number of Genes	Number of Plasmids	Genes per Plasmid
1S	4,884,062	51.82%	4550	3	93, 88, 3
2S	4,649,739	52.27%	4297	1	87
3S	4,884,418	51.82%	4557	3	92, 89, 1
4S	4,883,554	51.82%	4558	3	92, 88, 1
5S	4,881,291	52.16%	4508	1	87
6S	4,884,114	51.82%	4556	3	93, 88, 1
7S	4,883,922	51.82%	4551	2	93, 88
8S	4,964,964	50.53%	4698	-	-
9S	4,883,893	51.82%	4553	1	88
10S	4,886,993	52.07%	4624	3	91, 88, 48
12S	4,883,793	51.82%	4554	3	93, 88, 1
13S	4,884,338	51.82%	4537	4	87, 4, 3, 1
14S	4,884,314	51.82%	4553	3	92, 88, 3
15S	4,586,611	52.21%	4358	1	88
16S	4,882,966	51.79%	4554	-	-
17S	4,883,897	51.82%	4552	2	92, 88
19S	4,882,774	51.75%	4546	3	103, 49, 4
20S	4,964,860	50.51%	4693	-	-
22S	4,650,091	52.24%	4370	2	93, 87
23S	4,650,096	52.24%	4369	2	91, 87
24S	4,649,113	52.24%	4371	2	92, 87
25S	4,886,157	51.71%	4557	3	91, 87, 48

**Table 4:** Plasmids identified in the sequenced strains, with plasmid length (bp) and incompatibility group assignments.

Strain	AF598	AB232 (IncFII, IncX1)	AD529	AB241 (ColpVC)	AA974 (Col156)	AA474 (IncI1/B/O)	AA619 (IncX4)
1S	70,131	74,672	3126	-	-	-	-
2S	70,173	-	-	-	-	-	-
3S	70,142	74,654	-	2223	-	-	-
4S	70,113	74,695	-	2228	-	-	-
5S	70,103	-	-	-	-	-	-
6S	70,160	74,634	-	2257	-	-	-
7S	70,087	74,677	-	-	-	-	-
8S	-	-	-	-	-	-	-
9S	70,114	-	-	-	-	-	-
10S	70,103	74,655	-	-	-	-	32,112
12S	70,174	74,677	-	2240	-	-	-
13S	70,090	-	3144	2234	4648	-	-
14S	70,187	74,622	3125	-	-	-	-
15S	70,115	-	-	-	-	-	-
16S	-	-	-	-	-	-	-
17S	70,137	74,674	-	-	-	-	-
19S	-	-	-	-	4597	92,347	32,165
20S	-	-	-	-	-	-	-
22S	70,115	74,724	-	-	-	-	-
23S	70,109	74,724	-	-	-	-	-
24S	70,143	74,746	-	-	-	-	-
25S	70,111	74,684	-	-	-	-	32,105

**Table 5:** Results of the identification of R-M operons in the sequenced genomes using the DefenseFinder program.

Strain	Type I	Type II	Type IIG_2	Type III	Type IV
1S	1	0	0	1	0
2S	1	0	0	1	0
3S	1	0	0	1	0
4S	1	0	0	1	0
5S	1	1	0	1	0
6S	1	0	0	1	0
7S	1	0	0	1	0
8S	1	0	0	0	2
9S	1	0	0	1	0
10S	1	2	1	1	0
12S	1	0	0	1	0
13S	1	0	0	1	0
14S	1	0	0	1	0
15S	0	1	0	1	0
16S	1	0	0	1	0
17S	1	0	0	1	0
19S	1	0	0	1	0
20S	1	0	0	0	2
22S	1	0	0	1	0
23S	1	0	0	1	0
24S	1	0	0	1	0
25S	1	2	1	1	0

## Data Availability

The sequenced genomes were deposited at the NCBI under BioProject PRJNA1468550.

## Supplementary Materials

The following supporting information can be downloaded at: https://www.mdpi.com/article/10.3390/pathogens15101074/s1, Table S1: Numbers of virulence genes in the sequenced genomes with 0 indicating gene absence; Table S2: Numbers of antibiotic resistance genes and mutations in the sequenced genomes with 0 indicating gene absence.

## Abbreviations

The following abbreviations are used in this manuscript:

iNTS	invasive non-typhoidal *Salmonella*
MLST	Multi-Locus Sequence Typing
MTase	Methyltransferase
NTS	non-typhoidal *Salmonella*
R-M	Restriction-modification
SMRT	Single-Molecule Real-Time
